# Association Between Maternal Genome-Wide Polygenic Scores for Psychiatric and Neurodevelopmental Disorders and Adverse Perinatal Events: A Danish Population-Based Study

**DOI:** 10.1016/j.bpsgos.2025.100613

**Published:** 2025-09-17

**Authors:** Fenfen Ge, Yue Wang, Xiaoqin Liu, Trine Munk-Olsen, Kathrine Bang Madsen, Emil Michael Pedersen, Clara Albiñana, Esben Agerbo, Cynthia M. Bulik, Liselotte Vogdrup Petersen, Unnur A. Valdimarsdottir, Bjarni Jóhann Vilhjálmsson

**Affiliations:** a Centre of Public Health Sciences, Faculty of Medicine, University of Iceland, Reykjavík, Iceland; b The National Centre for Register-based Research, Department of Public Health, Aarhus University, Aarhus, Denmark; c Department of Clinical Medicine, Aarhus University, Aarhus, Denmark; d Department of Affective Disorders, Aarhus University Hospital – Psychiatry, Aarhus, Denmark; e Child and Adolescent Psychiatry Research Unit, Department of Clinical Research, University of Southern Denmark, Odense, Denmark; f Department of Medical Epidemiology and Biostatistics, Karolinska Institutet, Stockholm, Sweden; g Department of Psychiatry, School of Medicine, University of North Carolina at Chapel Hill, Chapel Hill, North Carolina; h Department of Nutrition, University of North Carolina at Chapel Hill, Chapel Hill, North Carolina; i Harvard T.H. Chan School of Public Health, Boston, Massachusetts; j Bioinformatics Research Centre, Aarhus University, Aarhus, Denmark; k Novo Nordisk Foundation Centre for Genomic Mechanisms of Disease, the Broad Institute of Massachusetts Institute of Technology and Harvard, Cambridge, Massachusetts

**Keywords:** Neonatal, Perinatal risk factors, Polygenic scores, Pregnancy, Psychiatric and Neurodevelopmental disorders

## Abstract

**Background:**

Phenotypic links between psychiatric disorders and adverse perinatal events are increasingly being reported, but the mechanisms remain unclear. In this study, we aimed to assess how polygenic scores (PGSs) for 8 psychiatric conditions influence perinatal risk.

**Methods:**

The main analysis included 13,085 mothers and their corresponding birth information. PGSs for psychiatric conditions were estimated using genome-wide association study data (excluding the iPSYCH cohort) via LDpred2 and used as exposures. Ten adverse perinatal events from Danish national registers served as outcomes. Associations were analyzed using logistic or multinomial regression, with false discovery rate correction applied.

**Results:**

We found that PGSs for psychiatric conditions were associated with heavy smoking (attention-deficit/hyperactivity disorder [ADHD], anxiety, and depression), lower likelihood of being overweight/obese (schizophrenia, anorexia nervosa, and obsessive-compulsive disorder [OCD]), very young maternal age (<20 years) at childbirth (ADHD, depression, and anxiety), and non-cohabitation (ADHD, schizophrenia, anxiety, and depression). Little evidence of an association between maternal PGSs for psychiatric conditions and birth weight, gestational age, and labor presentation was identified. We identified a novel dose-response relationship in which higher PGSs for ADHD, anxiety, and depression were associated with a greater cumulative burden of adverse perinatal events, whereas higher PGSs for anorexia nervosa and OCD were linked to a lower burden.

**Conclusions:**

High genetic liability for psychiatric conditions may partially explain the observed phenotypic associations between maternal mental illness and adverse perinatal events, with higher genetic liability generally associated with either an increase or decrease in the cumulative burden of adverse perinatal events in a dose-response–like manner.

## Plain Language Summary

Epidemiological studies have shown that women with psychiatric conditions face an increased risk of adverse perinatal events. However, the genetic mechanisms underlying these associations are not fully understood. We conducted a comprehensive study assessing the relationships between 8 genome-wide polygenic scores (PGSs) for psychiatric disorders (e.g., ADHD, depression, anxiety) and 10 adverse perinatal outcomes, as well as their cumulative burden. Results indicated that genetic risk was associated with certain adverse outcomes, such as lower likelihood of being overweight, very young maternal age, and noncohabitation. High genetic liability for psychiatric conditions may partially explain the phenotypic links between maternal mental illness and adverse perinatal events, emphasizing their potential in early identification of at-risk populations.

Evidence of phenotypic associations between psychiatric disorders and adverse perinatal events—among the leading causes of health issues for both mothers and newborns—continues to accumulate (1, 2, 3). Epidemiological studies based on large-scale prospective national health registers or nationwide cohorts have shown that women with preexisting or active psychiatric and neurodevelopmental disorders [e.g., schizophrenia (4), anorexia nervosa (5), obsessive-compulsive disorder (OCD) (6), bipolar disorder (7), depression (7,8), or autism spectrum disorder (ASD) (9)] are at increased risk of a variety of adverse pregnancy behaviors [e.g., more frequent smoking during pregnancy (4), and cesarean sections (10)] and neonatal outcomes [e.g., preterm birth (5, 6, 7), low Apgar score at 5 minutes (6,10), and low birth weight (7)]. However, the underlying mechanisms behind these associations remain inadequately understood, with unmeasured confounding by genetic or environmental factors potentially contributing to the observed associations.

Genetic factors play an important role in the development of mental illness, as shown in genome-wide association studies (GWASs) and twin studies (11,12). Specifically, estimated single nucleotide polymorphism (SNP)–based heritability on the liability scale is 8.9% for depression (13), 10% for anxiety (14), 11% to 17% for anorexia nervosa (15), and 24% for schizophrenia (16), with hundreds or thousands of genetic loci identified via GWASs. Polygenic scores (PGSs), which combine the weighted effect sizes of multiple genetic variants using well-established summary data, can be used to assess the genetic liability of an individual to specific mental illness, such as schizophrenia or depression (17). However, evidence on the associations between maternal genetic liability to mental illnesses and adverse perinatal events is lacking, likely due to the scarcity of maternal genetic information or well-documented perinatal factors (18, 19, 20). Moreover, previous studies have often lacked sufficiently long follow-up periods to assess the prepregnancy mental health history of mothers, limiting the ability to exclude potential phenotypic effects of already manifested mental illness and to disentangle these from the underlying influence of maternal genetic liability to psychiatric and neurodevelopmental disorders.

**Figure 1:**
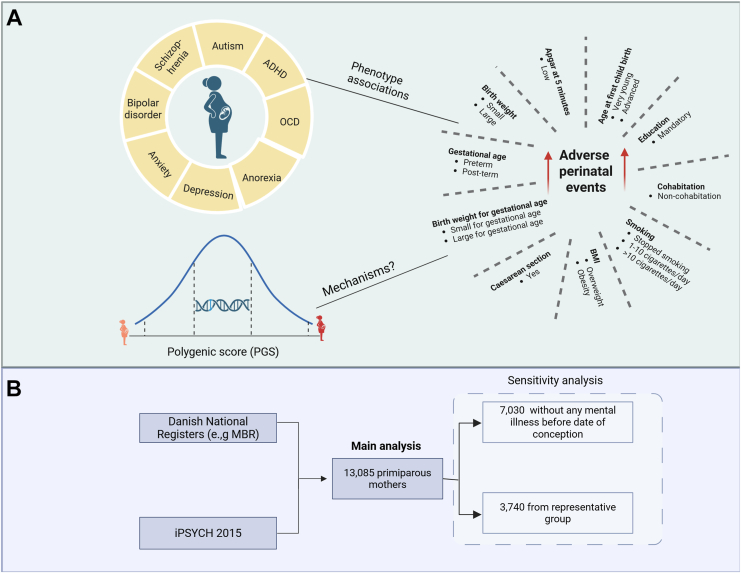
Study profile. **(A)** Study design. **(B)** Study sample. ADHD, attention-deficit/hyperactivity disorder; BMI, body mass index; MBR, Medical Birth Register; OCD, obsessive-compulsive disorder; PGS, polygenic score.

Leveraging individual genotyping data from the iPSYCH (Integrative Psychiatric Research) cohort and detailed pregnancy, delivery, and birth information from the Danish National Registers, we aimed to perform a comprehensive assessment of the associations between 8 genome-wide PGSs for psychiatric and neurodevelopmental disorders and 10 adverse perinatal events, as well as the cumulative burden of adverse perinatal events (Figure 1A). The primary analysis was conducted among all primiparous mothers available in the iPSYCH cohort (Figure 1B). To ensure the robustness and generalizability of our findings, 2 sensitivity analyses were performed. The first was restricted to individuals without preexisting mental illness, allowing us to examine the direct associations between PGSs and adverse perinatal events. The second was conducted in a representative subgroup to assess the generalizability of the findings.

## Methods and Materials

### Date Source and Study Population

We used data from the iPSYCH2015 (21), a population-based case-cohort sample that builds on the design of iPSYCH2012 (22). The iPSYCH2015 sample was selected from the Civil Registration System, which includes all singletons born between 1981 and 2008 who were alive and living in Denmark at 1 year of age with known maternal information. A total of 93,608 individuals diagnosed with one or more major psychiatric disorders were identified through the Danish Psychiatric Central Research Register and included in the case group. The anorexia nervosa samples were included from the ANGI (Anorexia Nervosa Genetics Initiative) (23) because they were samples within the same framework as iPSYCH2015. Moreover, a random sample of 50,615 individuals from the same birth cohort was selected to serve as a population-representative control group. DNA was extracted from blood samples collected at birth and stored as dried blood spots in the Danish Newborn Screening Biobank (24). Detailed information about iPSYCH2015 has been described in the cohort profile (21).

We focused on primiparous mothers because our previous study found that primiparity status influenced the risk of postpartum mental disorders (25). By linking the iPSYCH2015 cohort with national registers, we identified 13,085 such mothers of European ancestry with available genotypic data. Information on demographic characteristics (e.g., birth year or sex), medical diagnoses (e.g., ICD-10 code), prescriptions, and birth information were derived by cross-linkage with Danish national registers including the Medical Birth Register (MBR) (26), Psychiatric Central Research Register (27), National Prescription Registry (28), and Civil Registration System (29,30). Detailed descriptions of the registers used are shown in Table S1.

The current study obtained approval from the Danish Scientific Ethics Committee, the Danish Health Data Authority, the Danish Data Protection Agency, and the Danish Neonatal Screening Biobank Steering Committee.

### PGSs for Psychiatric and Neurodevelopmental Disorders

In the current study, among all individuals in the iPSYCH2015, we first excluded individuals who did not pass genotypic quality control, with minor allele frequency <0.01 and Hardy-Weinberg *p* value <10^−6^, and we restricted to the HapMap3 variants in the LDpred2 LD reference panel, resulting in 1,053,299 SNPs (31). We performed principal component (PC) analysis following Prive *et al.* (32) and obtained 20 PCs, and we defined genetically homogeneous individuals (i.e., same European ancestry) having <4.5 log distance units to the multidimensional center of the 20 PCs. The KING-relatedness robust coefficient was calculated, and individuals with a degree of relatedness greater than second-degree (>0.0884) were excluded, resulting in a final sample of 108,628 individuals for further analysis (33).

We generated PGSs for attention-deficit/hyperactivity disorder (ADHD), ASD, schizophrenia, depression, anxiety, bipolar disorder, OCD, and anorexia nervosa using the GWAS summary statistics from the Psychiatric Genomics Consortium (PGC), excluding the iPSYCH2015 sample, with the LDpred2 method (34). Detailed information about summary statistics was shown in our previous study (35) and Table S2. In a validation step, the PGSs for 8 psychiatric and neurodevelopmental disorders showed strong associations with the corresponding disorders in the iPSYCH2015 cohort, as measured by logistic regression adjusted for birth year, sex, and the first 10 PCs for population stratification (Table S3). The distribution of PGSs among the case and control groups is presented in Figure S1. Notably, individuals with the recorded psychiatric disorders of interest had higher PGSs compared with individuals without these disorders. The PGSs were standardized using *z* score transformations to have a mean of 0 and an SD of 1 within the study population.

### Measurements of Adverse Perinatal Events

#### Maternal Smoking and Body Mass Index During Pregnancy

Information on maternal smoking and body mass index (BMI) during pregnancy, collected at the first antenatal visit, is available in the MBR. Smoking status is categorized as nonsmoker, stopped smoking during pregnancy, smoking 1 to 10 cigarettes per day, and smoking >10 cigarettes per day. BMI (kg/m^2^) is classified into 3 categories: <25 (underweight and normal weight), 25 to 29.9 (overweight), and ≥30 (obesity).

#### Maternal Age at First Birth

Age at first birth was calculated by subtracting the date of birth of the first child, obtained from the MBR, from the mother’s date of birth, obtained from the Civil Registration System. Age at first birth was categorized as <20, 20 to 24, 25 to 29, and 30 to 36 years based on previous findings from our study indicating that very young, young, and advanced maternal age were associated with increased risk of psychiatric disorders in offspring (36).

#### Maternal Relationship and Education Status

Information on maternal relationship status (i.e., cohabiting with the offspring’s father or not) was obtained from the MBR, while data on maternal education during pregnancy (categorized as mandatory or above mandatory education) were extracted from the Civil Registration System.

#### Gestational Age and Birth Weight

Gestational age was estimated from the ultrasound scan; if unknown, 280 days were used as a replacement (37). It was then categorized as preterm (≤36 weeks), term (36–41 weeks), or post-term (>41 weeks) (36). We first classified birth weight as <2500 g, 2500 to 3999 g, and >3999 g. Then we calculated birth weights for gestational age using sex-specific reference curves for fetal growth (38) and categorized them into <10th percentile (small for gestational age), 10th to 90th percentile (normal for gestational age), and >90th percentile (large for gestational age).

#### Labor Presentation and Apgar Score

Cesarean section delivery was recorded in the MBR and categorized as either “with cesarean section (yes)” or “without cesarean section (no).” The 5-minute Apgar score was used as an indicator of neonatal condition and classified into 2 categories, 7 to 10 (normal) and <7 (low) (39).

### Covariates

Information on the calendar year of delivery was obtained from the MBR and treated as a continuous variable ranging from 1981 to 2008 to account for genotyping waves. The first 10 standardized genetic PCs were adjusted to account for population substructure in the genetic data, although the sample was restricted to individuals of European ancestry (22).

### Statistical Analysis

#### Main Analyses

Descriptive results are reported as frequencies and percentages. We assessed the associations between 8 types of PGSs and adverse perinatal events using logistic regression models for binary variables (e.g., maternal relationship status) or multinomial logistic regression models for categorical variables (e.g., maternal age at first birth). A post hoc multinomial logistic regression analysis was conducted to examine the associations between 8 PGSs and the cumulative number of adverse perinatal events. A p-trend was estimated by treating the number of adverse perinatal events as a continuous variable. The results were represented as odds ratios (ORs) and 95% CIs, and the effect estimates are interpreted as the change in odds for per unit (1-SD) increase in specific PGSs.

#### Sensitivity Analyses

First, to examine the direct associations between PGSs and adverse perinatal events, we conducted sensitivity analyses in a subgroup of mothers without any psychiatric disorders. Specifically, among the 13,085 primiparous mothers, we excluded those with any recorded psychiatric disorders (ICD-10 F codes) or prescriptions for antidepressants (ATC code N06A) from their own birth until 6 months prior to conception. This resulted in a final analytic sample of 7030 mothers. Because the population control group was randomly selected from the entire Danish population (22), we re-ran the analyses restricted to primiparous mothers within the representative population group to obtain estimates more reflective of the national population. We also performed a negative control analysis using a left-handedness PGS because left-handedness has not been associated with any adverse perinatal events (40).

#### Multiple Correction

False discovery rate corrections were applied to all tests (41), including the main analyses examining associations between maternal genome-wide PGSs for psychiatric and neurodevelopmental disorders and adverse perinatal events (number of tests, k = 144), the secondary analysis using the cumulative perinatal risk score as the outcome (k = 32), and the sensitivity analyses among mothers without a diagnosed prepregnancy mental illness (k = 144) and among those within the representative population group (k = 144).

All data analyses were conducted using R version 4.0 software.

## Results

### Descriptive Statistics

Descriptive characteristics for the study population are presented in Table 1. In total, 13,085 mothers were included in the main analysis, and the mean age at first birth was 25.1 years (SD = 3.9 years).

**Table 1:** Characteristics of Study Population

Characteristic	Main Analysis	Sensitivity Analyses	
	All Population, *N* = 13,085	Without Prepregnancy Mental Illness, *n* = 7030	Representative Population Group, *n* = 3740
Maternal Smoking			
Nonsmoker	8902 (68.0%)	4850 (69.0%)	2971 (79.4%)
Stopping smoking	877 (6.7%)	457 (6.5%)	195 (5.2%)
1–10 cigarettes/day	1937 (14.8%)	1007 (14.3%)	332 (8.9%)
>10 cigarettes/day	867 (6.6%)	450 (6.4%)	118 (3.2%)
Missing	434 (3.3%)	225 (3.2%)	111 (3.0%)
Maternal BMI During Early Pregnancy^a^			
Underweight and normal	7510 (57.4%)	4006 (57.0%)	2232 (59.7%)
Overweight	2641 (20.2%)	1383 (19.7%)	798 (21.3%)
Obesity	1877 (14.3%)	915 (13.0%)	493 (13.2%)
Missing	1057 (8.1%)	726 (10.3%)	217 (5.8%)
Maternal Age at First Birth			
<20 years	1012 (7.7%)	762 (10.8%)	146 (3.9%)
20–24 years	4902 (37.5%)	2756 (39.2%)	1064 (28.4%)
25–29 years	5281 (40.4%)	2656 (37.8%)	1843 (49.3%)
30–36 years	1890 (14.4%)	856 (12.2%)	687 (18.4%)
Maternal Cohabitation			
Cohabitation	10,751 (82.2%)	5789 (82.3%)	3355 (89.7%)
Non-cohabitation	2311 (17.7%)	1231 (17.6%)	385 (10.3%)
Maternal Education			
Above mandatory education	7381 (56.4%)	4117 (58.6%)	2846 (76.1%)
Mandatory education	5625 (43.0%)	2870 (40.8%)	882 (23.6%)
Missing	79 (0.6%)	43 (0.6%)	12 (0.3%)
Birth Weight for Gestational Age, Percentile			
Small for gestational age, <10th	1496 (11.4%)	891 (12.7%)	520 (13.9%)
Normal for gestational age, 10th–90th	10,070 (77.0%)	5377 (76.5%)	2826 (75.6%)
Large for gestational age, >90th	1290 (9.9%)	649 (9.2%)	328 (8.8%)
Missing	229 (1.8%)	113 (1.6%)	66 (1.8%)
Birth Weight			
<2500 g	713 (5.4%)	375 (5.3%)	178 (4.8%)
2500–3999 g	10,556 (80.7%)	5661 (80.5%)	3006 (80.4%)
>3999 g	1587 (12.1%)	881 (12.5%)	490 (13.1%)
Missing	229 (1.8%)	113 (1.6%)	66 (1.8%)
Gestational Age^b^			
Preterm	791 (6.0%)	414 (5.9%)	195 (5.2%)
Term birth	10,859 (83.0%)	5761 (81.9%)	3060 (81.8%)
Post-term	1435 (11.0%)	855 (12.2%)	485 (13.0%)
Apgar Score at 5 minutes^c^			
Normal	12,685 (96.9%)	6839 (97.3%)	3642 (97.4%)
Low	144 (1.1%)	69 (1.0%)	26 (0.7%)
Missing	256 (2.0%)	122 (1.7%)	72 (1.9%)
Cesarean Section			
No	10,677 (81.6%)	5786 (82.3%)	3089 (82.6%)
Yes	2408 (18.4%)	1244 (17.7%)	651 (17.4%)
Cumulative Adverse Perinatal Events			
0	1333 (10.2%)	726 (10.3%)	568 (15.2%)
1	2474 (18.9%)	1365 (19.4%)	941 (25.2%)
2	2762 (21.1%)	1454 (20.7%)	849 (22.7%)
3	2766 (21.1%)	1447 (20.6%)	665 (17.8%)
≥4	3750 (28.7%)	2038 (29.0%)	717 (19.2%)

Values are presented as *n* (%). In accordance with Danish data protection regulations, subcategories with few observations within certain variables (e.g., maternal smoking and maternal cohabitation) were not reported.

BMI, body mass index.

a Underweight and normal (<25); overweight (25–29.9); obesity (≥30).

b Preterm (≤36 weeks); term birth (36–41 weeks); post-term (>41 weeks).

c Low Apgar score was defined as Apgar score < 7.

### Maternal Smoking and BMI During Pregnancy

**Figure 2:**
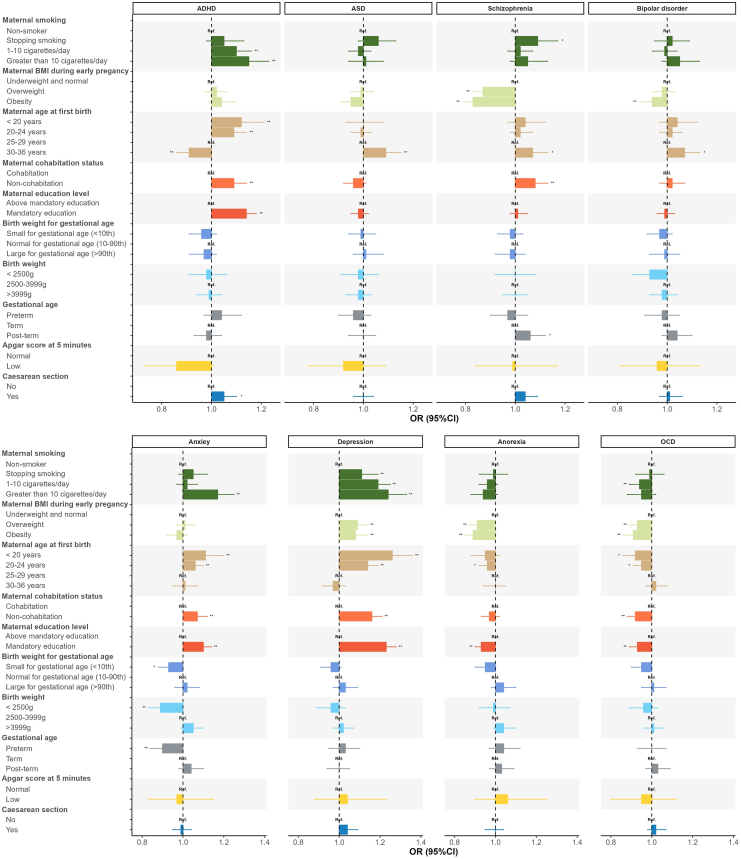
Associations between psychiatric conditions and adverse perinatal events. ∗*p* < .05. ∗∗False discovery rate–adjusted *p* < .05. ADHD, attention-deficit/hyperactivity disorder; ASD, autism spectrum disorder; OCD, obsessive-compulsive disorder; OR, odds ratio.

ADHD, anxiety, and depression PGSs were associated with maternal smoking of more than 10 cigarettes per day compared with nonsmokers, whereas the OCD PGS was negatively associated with smoking 1 to 10 cigarettes per day. The depression PGS was also associated with smoking cessation during pregnancy compared with nonsmoking mothers. The schizophrenia, anorexia nervosa, and OCD PGSs were negatively associated with overweight and obesity, while the bipolar disorder PGS was negatively associated with obesity only. The depression PGS was positively associated with both overweight and obesity during pregnancy (Figure 2 and Table S4).

### Maternal Age at First Birth

Each 1-SD increase in the ASD, schizophrenia, and bipolar disorder PGSs was associated with a higher risk of advanced age at first birth (30–36 years) compared with the 25 to 29 years group, although the associations for schizophrenia and bipolar disorder PGSs were reduced to nonsignificant after multiple testing correction. Higher genetic risk for ADHD, anxiety, and depression was associated with very young (<20 years) and young age (20–24 years) at first birth, whereas higher genetic risk for anorexia nervosa and OCD was negatively associated with young age at first birth but not after multiple testing correction.

### Maternal Relationship and Education Status

PGSs for ADHD, schizophrenia, anxiety, and depression were associated with increased risk of non-cohabitation during pregnancy, whereas higher genetic liability for OCD was associated with a reduced risk of non-cohabitation. Similarly, higher genetic risk for OCD and anorexia nervosa was associated with a lower risk of low educational attainment, while genetic risk for ADHD, anxiety, and depression was associated with an increased risk of low educational attainment.

### Gestational Age and Birth Weight

A positive association between the schizophrenia PGS and post-term birth was observed before multiple correction. Negative associations were found between the anxiety PGS and low birth weight (<2500 g), preterm birth, and small for gestational age (which did not survive multiple correction).

### Labor Presentation and Apgar Score

Polygenic risk for ADHD was associated with an increased risk of cesarean section before multiple correction. No associations were found between the 8 PGSs and Apgar score at 5 minutes.

### Cumulative Burden of Adverse Perinatal Events

**Figure 3:**
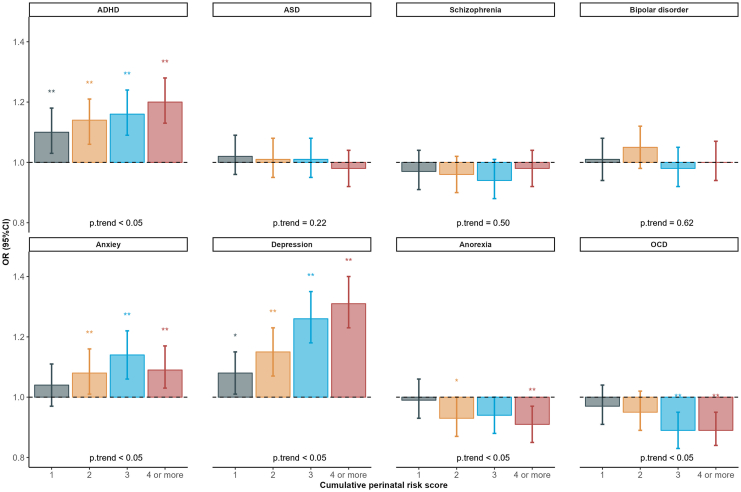
Associations between psychiatric condition polygenic scores and cumulative perinatal risk score. Reference: 0 adverse perinatal events. ∗*p* < .05. ∗∗false discovery rate–adjusted *p* < .05. ADHD, attention-deficit/hyperactivity disorder; ASD, autism spectrum disorder; OCD, obsessive-compulsive disorder; OR, odds ratio.

A dose-response association was observed between higher PGSs for ADHD, anxiety, depression, and the number of adverse perinatal events. For the ADHD PGS, ORs ranged from 1.10 for 1 event to 1.20 for 4 or more events. ORs ranged from 1.04 to 1.14 for the anxiety PGS and from 1.08 to 1.31 for the depression PGS. However, this pattern reversed with higher genetic risk for anorexia nervosa and OCD, which was associated with a lower likelihood of experiencing multiple adverse perinatal events (Figure 3).

Largely comparable results were observed among mothers without any mental illness (Table S5, Figure S2) and among the representative population group (Table S6, Figure S3). As expected, no statistically significant associations were observed between negative control exposure (PGS for left-handedness) and any adverse perinatal events (Table S7).

## Discussion

In this study, we examined the associations between maternal genetic liability—captured by 8 specific PGSs—and adverse perinatal events. Of the many findings presented, we highlighted two general trends. First, observed phenotypic associations seem to be partially driven by genetic risk for mental illness, although the direction of association between PGSs and adverse perinatal outcomes varies by disorder. For example, the schizophrenia, anorexia nervosa, and OCD PGSs were associated with a lower likelihood of overweight/obesity, whereas the depression PGS was associated with an increased risk. Second, we identified a dose-dependent relationship between higher PGSs for ADHD, anxiety, and depression and greater cumulative burden of adverse perinatal events. However, this pattern reversed for the anorexia nervosa and OCD PGSs, which were associated with a lower cumulative burden. These results remained comparable among mothers without formally diagnosed psychiatric conditions.

We extended prior studies that treated smoking as a binary variable (i.e., smoker vs. nonsmoker) (18,19) by examining smoking intensity and found that mothers with high genetic risk for ADHD, anxiety, and depression were more likely to smoke heavily during pregnancy. In contrast to epidemiological studies reporting high smoking prevalence among individuals with schizophrenia, bipolar disorder, and OCD (42), no association was observed between schizophrenia or bipolar disorder PGSs and smoking, and the OCD PGS was negatively associated with smoking. Further investigation of this paradoxical pattern may inform novel intervention strategies for both traits.

Previous studies have reported a negative association between schizophrenia PGSs and BMI in children (43) and have identified shared genetic loci between BMI and depression in a nonpregnant general population sample (44) using linkage disequilibrium score regression (LDSC). In our study, we confirmed the negative association between the schizophrenia PGS and BMI during early pregnancy and further examined the association between the depression PGS and BMI, providing complementary evidence from a PGS perspective. Although increasing evidence suggests higher rates of overweight/obesity in individuals with mental illnesses, such as bipolar disorder and OCD (45), we observed negative associations with the PGSs for these disorders, suggesting that elevated obesity rates in these populations may result from consequences of the disorder or medication effects (e.g., mood stabilizers, antidepressants, or antipsychotics) (46).

Furthermore, our results suggest that maternal age at first childbirth is influenced by PGSs, with distinct patterns across different psychiatric conditions. Extending previous findings that linked depression to younger maternal age at first birth, we found that higher genetic liability for autism was associated with older maternal age, whereas higher PGSs for anorexia nervosa and OCD were associated with lower risk of younger age at first birth. Expanding on previous studies using both LDSC (47) and Mendelian randomization (MR) analyses (48), we found that the associations persisted even among mothers with no diagnosed prepregnancy mental illness, suggesting a direct effect of maternal genetic liability to adverse perinatal events. For example, one study suggested associations between age at first birth and depression using univariable MR (49), although another study did not totally support these findings based on multivariable MR when considering age at first sexual intercourse, first birth, last birth, and age at menopause (50). Compared with methods that utilize summary data from GWASs (e.g., LDSC or MR) to calculate genetic correlations, associations derived from PGSs offer specific indices relevant to etiology, potentially improving the utility of PGS testing in clinical settings (51).

We found little evidence of an association between maternal PGSs for psychiatric conditions and birth weight or gestational age, which is consistent with previous findings from the Nurses’ Health Study 2 (18). Similarly, a study using data from the ALSPAC (Avon Longitudinal Study of Parents and Children) cohort (52) suggested that neither maternal nor child genetic liability for neurodevelopmental disorders (i.e., ADHD and ASD) was associated with birth weight and preterm delivery. One possible explanation is that birth weight is influenced by a combination of fetal, maternal, and paternal genetics (53). Moreover, gene-environment correlations may occur across generations if psychiatric disorders in the parental generation contribute to adverse childhood environments for the offspring (54). Another possible explanation involves medication effects, such as the use of psychotropic medications, which may explain the discrepancy between observed phenotypic associations and absent genetic associations. For example, several previous meta-analyses have found that the use of antidepressants during pregnancy is associated with an increased risk of preterm birth and low birth weight regardless of whether the comparison group consisted of all unexposed mothers or only depressed mothers without antidepressants (55, 56, 57).

### Implications

While PGSs cannot currently predict which individuals are at highest risk for experiencing specific perinatal complications (58), they hold potential for improving risk stratification when integrated with clinical and molecular data. Importantly, we observed that PGSs for psychiatric conditions were associated with adverse perinatal events even in the absence of a formal psychiatric diagnosis, highlighting their potential utility in early identification of at-risk populations.

The lack of evidence between maternal PGSs and birth weight or gestational age suggests that disentangling the genetic and environmental contributions to these traits likely requires family-based cohorts and genetically informed designs, such as sibling comparisons (59).

Our findings contribute to the growing body of evidence indicating that observational associations between adverse perinatal events and psychiatric disorders in offspring may be partially explained by shared genetic liability (60). Specifically, women with a higher polygenic burden for psychiatric conditions may be more likely to encounter adverse perinatal events and transmit genetic risk to their children, creating a spurious association between maternal perinatal exposures and offspring psychiatric outcomes.

### Strengths and Limitations

To the best of our knowledge, this is the first study to comprehensively explore the associations between 8 types of psychiatric and neurodevelopmental PGSs and well-documented adverse perinatal events among primiparous mothers. Another major strength of our study is the restriction of analyses to mothers without any preexisting mental illnesses, which allows for direct exploration of whether associations between maternal mental illnesses and adverse perinatal events are driven by genetic liability.

There are several limitations that need to be considered. First, comparing associations between psychiatric PGSs and adverse perinatal events remains challenging because these associations may reflect not only direct effects of genetic liability but also the influence of subclinical symptoms, pleiotropic effects, or other underlying pathophysiological mechanisms. Furthermore, differences in heritability, polygenicity, and GWAS sample sizes across psychiatric disorders, as well as the inclusion of individuals with psychiatric comorbidities in the GWAS samples, may limit the comparability of association strengths. For depression and bipolar disorder, we did not use the most recent GWAS summary statistics, which might have limited the explanatory power of the corresponding PGSs. Most of the associations identified between maternal exposure PGSs and offspring outcomes were of small magnitude and nonsignificant. Given that PGSs capture only a limited proportion of heritable variance, particularly for complex neurodevelopmental conditions such as ADHD, these estimates do not fully reflect the underlying genetic liability (61). Consequently, adjusting solely for maternal PGSs in observational studies is unlikely to sufficiently account for observed associations without complementary familial or sibling-based analyses. Future research should incorporate triangulation using multiple genetically informative designs, such as within-family MR, sibling comparisons, and children-of-twins designs (62). While our study identifies associations between PGSs and pregnancy-related risk factors, it does not infer causal relationships between maternal genetic liability and these factors. Another limitation is that all PGSs were derived from GWASs of individuals of European ancestry and applied to European cohorts, which may limit the generalizability of our findings to other populations.

In addition, the iPSYCH2015 cohort includes individuals born between 1981 and 2008, with the MBR updated until 2017. As a result, our study primarily captures relatively young mothers, which might have led to systematic differences in the associations between genetic risk for mental illness and the outcomes tested. Given the predominance of younger mothers in the cohort, some might not yet have reached the age at which the risk of developing certain conditions (e.g., gestational diabetes and preeclampsia) becomes pronounced. Finally, maternal smoking during pregnancy was self-reported in our study, and we used a detailed classification based on the number of cigarettes smoked per day. While this provides more detailed information, it may introduce information bias compared with a simpler yes or no classification. It is also important to note that maternal BMI was assessed during the early stage of pregnancy.

### Conclusions

In a large cohort covering a total of 13,085 primiparous mothers, we found that genetic risk may partially account for previously identified associations between maternal psychiatric conditions and adverse perinatal events, even in the absence of clinically diagnosed disorders. A dose-response relationship was identified in which higher PGSs for ADHD, anxiety, and depression were associated with a greater cumulative burden of adverse perinatal events, whereas higher anorexia nervosa and OCD PGSs were associated with a lower burden.

## Supplementary Material

Figure S1–S3 and Tables S1–S7

Key Resources Table
